# Validation of an Indirect Immunofluorescence Assay and Commercial Q Fever Enzyme-Linked Immunosorbent Assay for Use in Macropods

**DOI:** 10.1128/jcm.00236-22

**Published:** 2022-06-02

**Authors:** Anita Tolpinrud, John Stenos, Anne-Lise Chaber, Joanne M. Devlin, Catherine Herbert, An Pas, Magdalena Dunowska, Mark A. Stevenson, Simon M. Firestone

**Affiliations:** a Asia Pacific Centre for Animal Health, Melbourne Veterinary School, The University of Melbourne, Parkville, Victoria, Australia; b Australian Rickettsial Reference Laboratory, University Hospital Geelong, Geelong, Victoria, Australia; c School of Animal and Veterinary Sciences, The University of Adelaide, Roseworthy, South Australia, Australia; d School of Life and Environmental Sciences, The University of Sydney, Camperdown, New South Wales, Australia; e New Zealand Centre for Conservation Medicine, Auckland Zoo, Auckland, New Zealand; f School of Veterinary Science, Massey University, Palmerston North, New Zealand; Jockey Club College of Veterinary Medicine

**Keywords:** macropods, sensitivity, specificity, ELISA, Q fever, immunofluorescence assay, Bayesian latent class models, *Coxiella burnetii*, enzyme-linked immunosorbent assay, test validation

## Abstract

Kangaroos are considered to be an important reservoir of Q fever in Australia, although there is limited knowledge on the true prevalence and distribution of coxiellosis in Australian macropod populations. Serological tests serve as useful surveillance tools, but formal test validation is needed to be able to estimate true seroprevalence rates, and few tests have been validated to screen wildlife species for Q fever. In this study, we modified and optimized a phase-specific indirect immunofluorescence assay (IFA) for the detection of IgG antibodies against Coxiella burnetii in macropod sera. The assay was validated against the commercially available ID Screen Q fever indirect multispecies enzyme-linked immunosorbent assay (ELISA) kit (IDVet, Grabels, France) to estimate the diagnostic sensitivity and specificity of each assay, using Bayesian latent class analysis. A direct comparison of the two tests was performed by testing 303 serum samples from 10 macropod populations from the east coast of Australia and New Zealand. The analysis indicated that the IFA had relatively high diagnostic sensitivity (97.6% [95% credible interval [CrI], 88.0 to 99.9]) and diagnostic specificity (98.5% [95% CrI, 94.4 to 99.9]). In comparison, the ELISA had relatively poor diagnostic sensitivity (42.1% [95% CrI, 33.7 to 50.8]) and similar diagnostic specificity (99.2% [95% CrI, 96.4 to 100]) using the cutoff values recommended by the manufacturer. The estimated true seroprevalence of C. burnetii exposure in the macropod populations included in this study ranged from 0% in New Zealand and Victoria, Australia, up to 94.2% in one population from New South Wales, Australia.

## INTRODUCTION

Q fever, or “query fever,” is a zoonotic disease of global public health importance that is caused by the intracellular bacterium Coxiella burnetii (1). The disease was first described in Australia in 1937 and occurs worldwide, with the notable exception of New Zealand (1–3). Coxiella burnetii benefits from a marked lack of host specificity and has been demonstrated in a wide range of vertebrate and arthropod hosts worldwide, including a variety of terrestrial and marine mammals, birds, reptiles, and amphibians (4–7). In humans, the disease usually manifests as an acute flu-like illness, sometimes with chronic and potentially life-threatening sequelae. Although most commonly maintained and transmitted by ruminant livestock, where it causes abortions and considerable economic losses, wild animals and ticks are thought to be important reservoirs for infection (1, 8).

In Australia, macropod species (kangaroos and wallabies) have been implicated as the main wild animal reservoir of C. burnetii, with seroprevalence rates upward of 40% identified in some areas (9–13). A recent survey of kangaroo meat intended for pet consumption showed that 29% of the tested food packets (*n *= 58) were positive for C. burnetii by PCR assay, indicating that active infections may be widespread in these species (14). Coxiella burnetii DNA has also been detected in up to 12% of macropod feces, suggesting that fecal shedding and environmental contamination may be an important transmission route (10, 13). Additionally, direct or indirect contact with macropods has been identified as a likely risk factor for human cases of Q fever (15–17). Macropods are thought to carry C. burnetii without clinical signs, although very little is known about the pathophysiological and immunological responses of these species to the organism (10, 13).

The diagnosis of coxiellosis in wildlife is usually achieved through demonstration of the presence of serum antibodies or molecular detection of C. burnetii DNA in tissues or excretions. However, few diagnostic tests have been formally validated in wildlife species, and the diagnostic accuracy of the available tests as applied to the various species is largely unknown. While molecular techniques such as PCR are considered highly sensitive tools for detecting C. burnetii infections in animal tissues (8), their use in epidemiological investigations is complicated by uncertainties surrounding species-specific differences in pathophysiological processes, such as routes and duration of excretion and tissue trophism. These processes are poorly characterized in most species, although ruminant and experimental animal models suggest that they are highly variable and unpredictable between species, often with a relatively short-lived active infection and intermittent, unpredictable shedding periods (18–21). In contrast, although species-specific information on antibody response and duration of immunity is rarely available, antibodies tend to persist in circulation for a prolonged period, and animals may seroconvert without actively shedding C. burnetii (22). Serological techniques aimed at detecting antibodies to C. burnetii would therefore be more sensitive methods to detect past or present exposure of a population to the organism. Although it is a measure of historical exposure rather than current infection, serology is an invaluable tool for disease surveillance, especially in wildlife populations, and can be used to demonstrate possible epidemiological links *post hoc* in outbreak investigations, even if the animal has already cleared the infection (23–25).

Coxiella burnetii exists in two antigenically distinct phase variations (phase I and phase II), both of which are used in the development of serological assays. The most commonly used serological techniques available for coxiellosis are the enzyme-linked immunosorbent assay (ELISA), the indirect immunofluorescent assay (IFA), and the complement fixation test (CFT) (3). The use of CFT in macropod species is limited due to its poor sensitivity and frequent nonspecific reactions, rendering test results uninterpretable (10, 26). The IFA has the advantages of being highly sensitive, phase specific, and cost-effective and is considered the reference method for the diagnosis of Q fever in humans (1). While it has been successfully adapted for use in a range of other species, there are no published reports of its use in macropods (3, 26–30). However, the IFA is more laborious to perform, compared with the ELISA, and is somewhat subjective to read, relying on a skilled technician for accurate interpretation. ELISAs are commonly preferred for the screening of large numbers of samples because they are relatively quick and easy to perform and the results are less likely to be affected by interpretation errors (1). The only multispecies ELISA kit commercially available in Australia (the ID Screen Q fever indirect multispecies ELISA kit; IDVet) has not been evaluated for its accuracy when used on serum from marsupials, although it has been shown to have good diagnostic accuracy in a range of other species, including rodents, lagomorphs, and carnivores (31, 32). A number of custom in-house ELISA protocols for use in macropods have been described in the literature; however, these have not been comprehensively validated (10, 11).

A formally validated, convenient, and affordable serological test for coxiellosis in macropods could help improve our understanding of the role of these species in the ecology of Q fever in Australia and would be a highly useful tool in national Q fever surveillance and epidemiological studies. With this background, the objective of this study was to validate two serological tests for the detection of C. burnetii antibodies in macropod serum for these purposes. A phase-specific IFA was developed and optimized specifically for the detection of IgG antibodies against C. burnetii in macropod serum. Given the absence of an accepted gold standard for C. burnetii exposure, this IFA was evaluated against a commercially available multispecies ELISA using Bayesian latent class analysis to estimate the diagnostic sensitivity and specificity of each assay, in accordance with the OIE recommendations and the standards for the reporting of diagnostic accuracy studies that use Bayesian latent class models (33, 34).

## MATERIALS AND METHODS

### Samples.

This was a retrospective study of serum samples collected from 303 macropods. Of these, 85 were opportunistically collected from hunted eastern gray kangaroos (Macropus giganteus [*n *= 62]) and red kangaroos (Osphranter rufus [*n *= 23]) at two separate locations in South West Queensland, Australia, in May 2019 and July 2020. Fifty of these animals also had tissue samples collected from multiple organs and tested for the presence of C. burnetii DNA by PCR assay, using three different PCR targets (IS*1111*, *com1*, and *htpAB*), as part of a different (unpublished) study. The results of that study were made available to help with the selection of a suitable positive control sample. Archived serum samples from eastern gray kangaroos that had been collected from a suburban population in Anglesea, Victoria, Australia, between 2015 and 2019 (*n = *30) and from six distinct populations in eastern New South Wales, Australia, between 2005 and 2019 (*n *= 158) were also made available for the study (35, 36). Those samples had been collected for other purposes, and the exposure of those populations to C. burnetii was unknown prior to inclusion in this study. The geographical locations of the origins of the Australian samples are shown in Fig. S1 in the supplemental material.

Additionally, 30 red-necked wallaby (Notamacropus rufogriseus) serum samples were obtained from New Zealand to serve as known negative controls. Of those, 20 had been collected postmortem from free-ranging wallabies that had been euthanized as part of a pest control program in Mackenzie Pass, while 10 had been collected from captive wallabies at Auckland Zoo during routine health checks between 2016 and 2019. All serum samples had been separated from whole blood and stored at −20°C prior to inclusion in the study.

### ELISA.

All macropod sera were tested in duplicate using the ID Screen Q fever indirect multispecies ELISA kit (IDVet, Grabels, France) following the manufacturer’s instructions. The ID Screen ELISA uses phase I and phase II C. burnetii antigens derived from a French isolate from an aborted bovine placenta to coat the microwells and a protein A- and protein G-based conjugate to detect IgG (31). Prior to testing with the ELISA, a qualitative assessment of the kit’s conjugate binding to serial dilutions of pooled sera from a range of marsupial species (diluted 1:50, 1:100, 1:200, and 1:400 in phosphate-buffered saline [PBS]) was performed by immunoblotting, using a conjugate dilution of 1:100 in PBS containing 0.05% Tween 20 and 5% (wt/vol) skim milk powder, and following the protocol previously described by Vaz et al. (37). The cutoffs recommended by the manufacturer were used for interpretation of the ELISA results, with a sample to positive ratio percentage (S/P%) of >50% being considered positive. The S/P% was calculated according to the manufacturer’s formula, where OD is the mean optical density of the two duplicates of the respective samples or controls, measured at 450 nm:

S/P%=ODsample − ODneg controlODpos control − ODneg control × 100

Samples that returned an equivocal S/P% result of 40% to ≤50% were repeated (*n = *15). If a sample crossed the positive cutoff threshold on the repeat test, it was reclassified as positive; otherwise, it was counted as negative for the purpose of subsequent analyses.

### IFA.

A macropod-specific IFA was developed and optimized for inclusion in the latent class model. Forty-well Teflon-coated microscope slides (Tekdon Inc., USA) were prepared following standard protocols at the Australian Rickettsial Reference Laboratory. Briefly, 40-well microscope slides were coated with phase I (1:20 working dilution) or phase II (1:15 working dilution) antigen (Virion\Serion, Würzburg, Germany) in PBS and air dried. The slides were then fixed in acetone for 2 min, air dried, and stored at −20°C until use. Fluorescein-labeled anti-kangaroo IgG was prepared and used to detect antibodies to C. burnetii in macropod test sera. IgG was purified from rabbit anti-kangaroo whole serum antibody (Bethyl Laboratories Inc., Montgomery, TX, USA) using a protein A-based antibody purification kit (ab109209; Abcam, Cambridge, MA, USA), according to the manufacturer’s instructions. The purified antibodies were then conjugated to fluorescein isothiocyanate (FITC) using the FITC Conjugation Kit (Fast) Lightning-Link (ab188285; Abcam), according to the manufacturer’s protocol.

For the assay, test sera and conjugate were diluted in 2% casein in PBS. A checkerboard titration was performed to determine the optimum conjugate concentration for each batch within the dilution range of 1:25 to 1:200. Macropod test sera were tested in duplicate at a 1:32 starting dilution for both phase I and phase II antibodies, and positive sera were subsequently titrated in serial 2-fold dilutions to determine the endpoint titer. The initial cutoff serum dilution was determined by testing all of the negative control sera in 2-fold serial dilutions, starting at 1:16, until all nonspecific fluorescence was eliminated. The assay was performed by spotting the diluted test sera onto the prepared slides and incubating them in a humid chamber for 40 min at 37°C before washing the slides in 10% PBS and allowing them to air dry. A known negative control sample and a positive-control sample were included on each slide. The positive control sample was selected based on the demonstration of strong fluorescence during an initial IFA screening in the early optimization process (endpoint titers of 1:4,096 for phase I antigen and 1:1,024 for phase II antigen), a high S/P% on the ELISA (147.8%), and PCR positivity for multiple organs and all three PCR targets. Diluted conjugate (1:200) was then spotted onto each well prior to a second incubation period, as described above. Slides were washed again, air dried, and mounted before being examined for fluorescence with an immunofluorescence microscope at ×40 magnification. Two technicians independently read the same set of slides for the initial screening of the first 118 samples in order to determine the interoperator reliability of the assay; both technicians were blinded to the identity of the samples.

### Statistical analyses.

A Bayesian latent class model was developed to estimate the diagnostic sensitivity and specificity of each test in the absence of a gold standard, assuming conditional dependence between the tests (38). Prior information about the diagnostic specificity and sensitivity of each assay was modeled using unimodal beta distributions based on published data. Here, we assumed a most likely diagnostic sensitivity and specificity of 94% and 92%, respectively, for the IFA and a most likely diagnostic sensitivity and specificity of 90% and 97%, respectively, for the IDVet ELISA (26, 31). To keep the priors vague, the minimum plausible value for the lower bound was assumed to be 30% for sensitivity and 35% for specificity for each test. These details were then used to derive parameters for beta prior distributions using the epi.betabuster function in the contributed epiR package (39, 40) in R (41). A similar approach was taken to develop prior probability distributions of the prevalence of C. burnetii exposure among macropods for each sampled population. Subject matter experts (*n *= 3) and published literature were consulted to obtain the most likely and maximum C. burnetii exposure prevalence rates for each of the study areas, with 95% confidence. Priors for Queensland and northern New South Wales were based on limited data from earlier macropod studies and human and animal case notifications (11), and prevalence was assumed to gradually reduce further south and close to major urban areas, such as Sydney (42). Mixture priors were used for the prior distribution of C. burnetii exposure prevalence in the New Zealand and Victorian populations, where Q fever is thought to be absent or only focally clustered, respectively (3, 43), to allow for the prior prevalence distribution for these two populations to have increased density over zero. A two-test, 10-population Bayesian latent class model was then implemented, with the assumption that there were differences in the true prevalence of C. burnetii exposure for macropods from each geographical location (40). A two-dependent-test, ≥3-population model is nonidentifiable due to the algebraic structure of the model (44) and not due to a lack of degrees of freedom. For this reason, the choice of priors for the Markov chain Monte Carlo method is important. If a model lacks identifiability, then the inclusion of informative priors can allow useful inferences to be drawn. Full details of the prior information incorporated in the model are listed in Table 1.

**TABLE 1:** Prior distributions used in the Bayesian latent class analysis

			Beta distribution		
Test or region^*a*^	Parameter	Mode (95% PI)^*b*^	Alpha	Beta	Reference or source
IFA	Sensitivity	0.94 (0.300–0.974)	2.608	1.103	26
IFA	Specificity	0.92 (0.35–0.972)	3.104	1.183	26
ELISA	Sensitivity	0.90 (0.299–0.968)	2.706	1.190	31
ELISA	Specificity	0.97 (0.349–0.979)	2.937	1.060	31
Sydney basin (NSW)	Prevalence	0.1 (0.035–0.788)	1.114	2.026	See text
Nelson Bay (NSW)	Prevalence	0.2 (0.050–0.950)	1.000	1.000	See text
Coffs Harbour (NSW)	Prevalence	0.2 (0.050–0.950)	1.000	1.000	See text
South West Queensland	Prevalence	0.25 (0.050–0.950)	1.000	1.000	11
Victoria	Presence	0.05 (0.017–0.200)^*c*^	2.063	21.197	43, 58
	Prevalence	0.12 (0.035–0.600)	1.394	3.889	
New Zealand	Presence	0.01 (0.004–0.100)^*c*^	1.335	34.165	3
	Prevalence	0.01 (0.004–0.100)	1.335	34.165	

a NSW, New South Wales.

b PI, prediction interval.

c Mixture priors were used to account for the possibility of zero prevalence in the New Zealand and Victorian populations. The first prior represents the probability of the population being infected, while the second models the prevalence if the population was indeed infected.

The lower and upper bounds for the covariance terms that model the conditional dependence between tests were adapted from the report by Dendukuri and Joseph (45). The dependence terms were specified as independent uniform distributions and Bayesian inferences were based on the joint posterior distribution using OpenBUGS (46) working through the R2OpenBUGS package (47) in R (41). The models were run using two independently initiated chains of 10,000 iterations, discarding the first 2,000 iterations as burn-in, based on visual assessment of convergence in plots of the chains using the mcmcplots package (48) in R, the Gelman-Rubin statistic (49), and estimates of effective sample size (>200 for all inferred parameters) and autocorrelation by lag. Final inferences were presented as the median point estimate and 95% credible interval (CrI) of the marginal posterior distributions for each of the unknown parameters.

To test the influence of the priors on the final model outputs, a prior sensitivity analysis was performed (see Table S1 in the supplemental material). Additionally, analyses excluding the samples from the 23 red kangaroos and 30 New Zealand wallabies were carried out to assess the impact of using sera from members of different genera of *Macropodidae* on the diagnostic sensitivity and specificity results. Finally, modeling was repeated using a range of different S/P% cutoffs for the ELISA, allowing a two-way receiver operating characteristic (ROC) curve to be created. This was used to estimate an optimum cutoff value for the highest combined diagnostic sensitivity (DSe) and diagnostic specificity (DSp) for the ELISA, as assessed using Youden’s index (J=DSe+DSp–1)) (50.

Cohen’s kappa statistic (κ) was calculated to assess the interoperator reliability of the IFA, by determining the level of agreement between the two technicians beyond that of chance alone (51). The calculation was performed using the epi.kappa function in epiR (39), and the statistic was interpreted as described by Altman (52), with κ values of >0.80 indicating very good agreement, 0.61 to 0.80 good agreement, 0.41 to 0.60 moderate agreement, 0.21 to 0.40 fair agreement, and ≤0.2 poor agreement.

## RESULTS

### Determination of ELISA conjugate suitability for use in macropod species.

The immunoblots performed with marsupial sera using the IDVet ELISA conjugate showed relatively good reactivity with all of the macropod species tested (see Fig. S2 in the supplemental material), confirming that the kit has the ability to detect both kangaroo and wallaby antibodies.

### Comparison of the diagnostic performance of the IDVet ELISA and the IFA.

**FIG 1:**
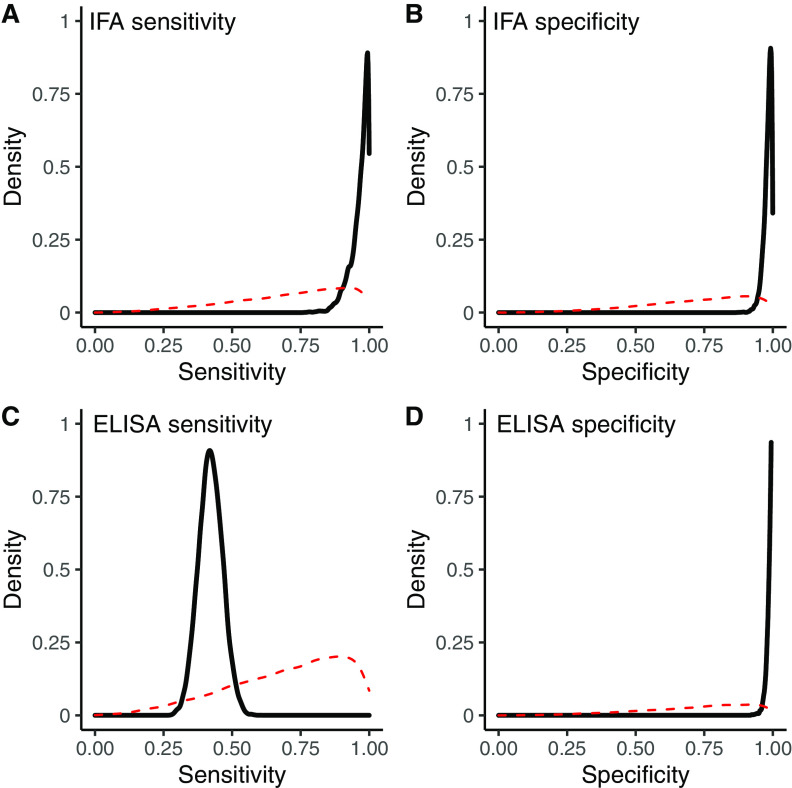
Prior and posterior distributions for the diagnostic sensitivity and specificity of the ELISA and IFA for detection of IgG antibodies against C. burnetii in macropod serum. The red dashed lines represent the prior distribution, while the solid black lines indicate the posterior distribution.

A total of 135 macropod samples (45%) were positive on the IFA, while 57 samples (19%) were positive on the IDVet ELISA. A detailed comparison of the results for each test, stratified by individual macropod population, is shown in Table 2. Fifteen test sera initially returned an equivocal result for the ELISA. After retesting, one sample was reclassified as positive (S/P% of 49.1 changed to 51.9), while the rest remained at ≤50% and were classified as negative. All of the samples that were positive on the ELISA were also positive on the IFA. Positive samples were identified among both red kangaroos (9/23 samples on ELISA and 10/23 samples on IFA) and eastern gray kangaroos (48/250 samples on ELISA and 125/250 samples on IFA) with both tests, while all of the negative control sera from New Zealand were negative on both the IFA and the ELISA. The posterior median estimates of the diagnostic sensitivity and specificity of the ELISA were 42.1% and 99.2%, respectively, while the IFA had posterior median estimates of 97.6% sensitivity and 98.5% specificity (Fig. 1; see Table 3 for CrIs). Removing the red kangaroos and New Zealand wallabies from the model produced no marked change in diagnostic sensitivity and specificity estimates (see Table S2). The robustness of our results with respect to choice of priors showed that the model was mainly informed by the data (Fig. 1; also see Table S2).

**TABLE 2:** Numbers of macropod samples included in the study and the relative test results for the IDVet Q fever ELISA and the IFA, stratified by geographical region

Region^*a*^	No.	No. with test results of:			
		IFA positive		IFA negative	
		ELISA positive	ELISA negative	ELISA positive	ELISA negative
Roma, Queensland	50	18	8	0	24
St. George, Queensland	35	4	10	0	21
Look At Me Now Headland, NSW	31	6	19	0	6
Arrawarra, NSW	20	5	4	0	11
Heritage Park, NSW	14	3	5	0	6
Nelson Bay, NSW	34	14	18	0	2
Sydney water catchment area, NSW	35	7	14	0	14
Western Sydney, NSW	24	0	0	0	24
Anglesea, Victoria	30	0	0	0	30
New Zealand	30	0	0	0	30
Total	303	57	78	0	168

a NSW, New South Wales.

**TABLE 3:** Bayesian estimates of the diagnostic sensitivity and specificity of the IDVet Q fever ELISA and the IFA when used on macropod sera

Parameter	ELISA (95% CrI)	IFA (95% CrI)	Conditional dependence^*a*^
Diagnostic sensitivity	0.421 (0.337–0.508)	0.976 (0.880–0.999)	
Diagnostic specificity	0.992 (0.964–1.000)	0.985 (0.944–0.999)	
*ρ_d_*			0.070 (−0.094 to 0.265)
*ρ_n_*			0.383 (0.012 to 0.899)

a The conditional dependence term is used to represent the correlation between the ELISA and IFA results for samples from animals inferred to have been diseased (*ρ_d_*) or nondiseased (*ρ_n_*).

### Determination of the optimum cutoff for the IDVet ELISA with macropod samples.

**FIG 2:**
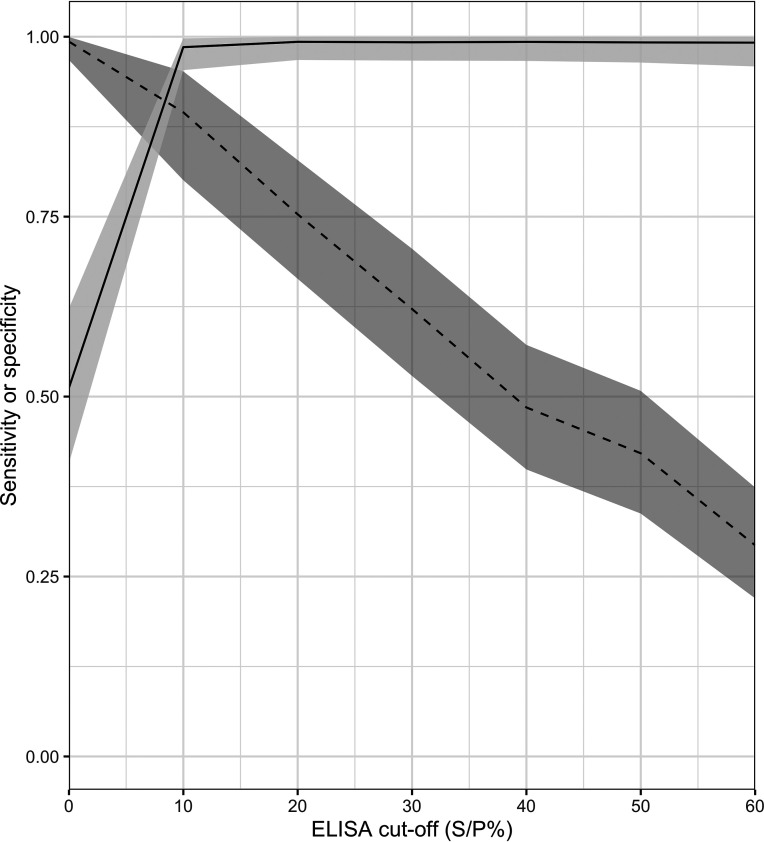
Diagnostic sensitivity and specificity with different cutoffs for the IDVet ELISA for macropod sera, estimated using Bayesian latent class analysis. The solid line represents specificity, the dashed line represents sensitivity, and the shading indicates the 95% CIs.

Given the low sensitivity and high specificity of the IDVet ELISA using the manufacturer-recommended cutoff, retesting across a range of cutoffs showed that the optimum cutoff (highest combined sensitivity and specificity, as determined by the Youden’s index) was at an S/P% value of 10% (Fig. 2). With this cutoff, the estimated diagnostic sensitivity was 89.5% (95% CrI, 80.1 to 95.2%), while the diagnostic specificity was 98.6% (95% CrI, 95.4 to 99.8%).

### Interoperator agreement for the IFA.

A total of 532 individual IFA wells were read by two independent technicians to assess the interoperator reliability of the test. Analysis of the interoperator agreement for the IFA showed a nearly perfect overall observed agreement of 98.7% (κ = 0.97 [95% CI, 0.89 to 1.0]).

### Phase variations and titrations.

**FIG 3:**
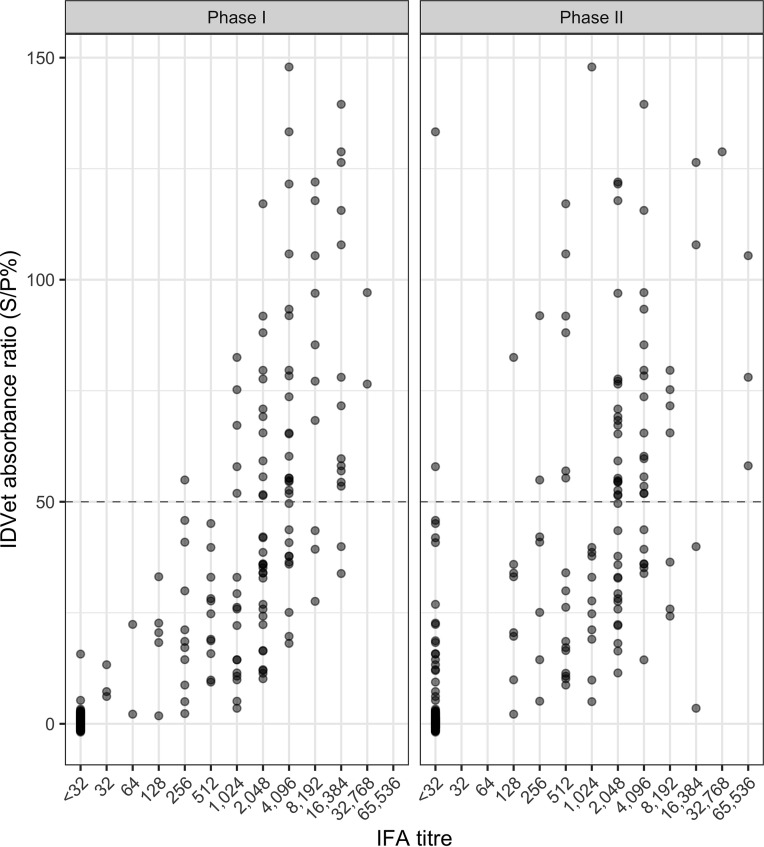
IFA phase I and phase II titers plotted against the absorbance ratio for the IDVet ELISA. Titers are expressed as the reciprocal of the highest endpoint dilution that produced immunofluorescence for each sample.

Of the 135 samples that were positive on the IFA, all were positive for phase I antibody, and 114 were also positive for phase II antibody. The phase I end titers ranged from 1:32 to 1:32,768, while the phase II end titers ranged from 1:128 to 1:65,536. Figure 3 shows the distribution of the IFA titers for phase I and II antigens and their relative relationship to the ELISA absorbance ratios, with some moderately high IFA titers for samples that were below the IDVet manufacturer-recommended cutoff for the ELISA (S/P% value of 50).

### Q fever seroprevalence in the included macropod populations.

The apparent seroprevalence in the tested macropod populations varied between tests and across geographical locations, ranging from 0% to 94.1% for the IFA and from 0% to 41.2% for the ELISA (Table 4). The highest seroprevalence estimates were from samples collected in Nelson Bay (the Hunter Region) and Look At Me Now Headland (Coffs Harbor) in New South Wales, while exposure to C. burnetii was not detected in the populations sampled in Victoria, New Zealand, and near Sydney (New South Wales), Australia.

**TABLE 4:** Apparent and estimated true seroprevalence rates of Q fever in the macropod populations included in the study, stratified by region

Region^*a*^	No.	Apparent sero-prevalence (%) by:		True seroprevalence (95% CrI) (%)
		IFA	ELISA	
Roma, Queensland	50	52	36	52.7 (38.1 to 67.4)
St. George, Queensland	35	40	19	40.5 (24.6 to 58.0)
Look At Me Now Headland, NSW	31	81	19	81.3 (64.0 to 94.8)
Arrawarra, NSW	20	45	25	45.7 (25.0 to 68.2)
Heritage Park, NSW	14	57	21	57.5 (31.5 to 81.2)
Nelson Bay, NSW	34	94	41	94.2 (82.3 to 99.6)
Sydney water catchment area, NSW	35	60	20	58.0 (41.2 to 74.2)
Western Sydney, NSW	24	0	0	3.1 (0.2 to 14.5)
Anglesea, Victoria	30	0	0	0 (0.0 to 0.0)
New Zealand	30	0	0	0 (0.0 to 0.0)

a NSW, New South Wales.

## DISCUSSION

While a few macropod-specific Q fever ELISAs have been developed and described in the literature, to the best of our knowledge none of them has been formally validated to quantify their diagnostic sensitivity and specificity (10, 11). There are a number of challenges related to diagnostic test validation for wildlife species, including access to reference positive and negative control samples representative of the target population(s) for which the test is being validated, the availability of sufficient numbers of samples to allow parameters to be estimated with certainty, the availability of a species-appropriate gold standard reference test, and detailed knowledge of species-specific pathophysiology and immunology (53, 54). In this study, we used a Bayesian latent class analysis to overcome the lack of known positive control samples, the absence of a gold standard reference test, and uncertainty regarding the true disease status of many of the individual animals sampled (38). The IFA developed for this study clearly outperformed the commercially available IDVet ELISA in terms of diagnostic sensitivity, with highly comparable diagnostic specificity. While a commercially available ELISA is arguably more convenient to carry out, our results show that using an ELISA could misclassify close to 60% of all truly diseased individuals in a sample set. Unless these performance characteristics could be improved, perhaps by altering the cutoff, it is recommended that future serosurveys for C. burnetii in macropods use the IFA where possible. This is in line with current recommendations for Q fever screening in humans, for which the IFA is considered the reference test (1).

The Bayesian estimates of the performance of the IDVet ELISA with different cutoffs show that the diagnostic sensitivity could be significantly improved by lowering the cutoff value from the manufacturer’s recommended S/P% value of 50% to 10%, while still maintaining a relatively high specificity. It also may be possible to further improve the ELISA’s sensitivity, for example, by optimizing the reagent or sample dilutions or by changing the conjugate. The choice of conjugate is an important consideration for diagnostic test development and performance and can present a particular challenge in less studied nondomestic species, particularly when the target species is evolutionarily distinct or lacks domestic counterparts. Vaz et al. (37) provide a good overview of the immunoglobulins in marsupials. They tested the binding efficiency of a range of potential conjugates against a variety of marsupial and monotreme sera and found that, overall, anti-kangaroo antibody was the most reliable in all marsupial species, with minimal observed differences in conjugate affinity across macropod species. Protein A was also reasonably effective in all species of wallabies and kangaroos but bound poorly to eastern bettong (Bettongia gaimardi), woylie (Bettongia penicillata), and koala (Phascolarctos cinereus) sera. Protein G, on the other hand, was largely ineffective against all marsupial species tested. The IDVet ELISA, based on a combined protein A and protein G conjugate, had an immunoblot profile similar to that of protein A alone (31, 37). It is possible that using an anti-kangaroo conjugate instead, similar to that of the IFA developed here, could help increase the sensitivity of the ELISA. However, because the main benefits of a commercially available ELISA kit include ease of use and rigorous quality control and because introducing changes to the manufacturer’s protocol is likely to affect the user friendliness or reproducibility of the assay, introducing such changes was considered beyond the scope of this study.

This study included sera from three different macropod species, with the assumption that there would be minimal differences in the diagnostic performance of the two assays among the members of *Macropodidae*. Sample inclusion was largely determined by availability, particularly with regard to the known negative controls, due to the existence of a limited number of known Q fever-free macropod populations. To test the possible effects of inclusion of the wallaby sera on our results, analyses were rerun omitting the New Zealand population and using the Victorian kangaroos as the low-prevalence population. The exclusion of the red kangaroo and New Zealand wallaby populations did not markedly change our results (see Table S2 in the supplemental material). For this reason, the data were included in the final model to increase the sample size and thus reduce the width of the CrIs around the diagnostic specificity estimates. While the combined result from the modeling and the conjugate testing would suggest that there is a negligible difference in diagnostic performance between macropod species, we cannot fully rule out the possibility that a species difference does, in fact, exist. We conclude that, for the populations of macropods included in this study, these differences were too small to detect, given the number of samples available for analysis.

One benefit of the IFA is that it can be used to determine the phase positivity and relative titers to phase I and phase II antigens. In humans, phase II is associated with acute Q fever, with phase II-specific IgG antibodies appearing earlier and persisting at higher titers than phase I antibodies (55). A similar antibody response has also been demonstrated in mice and goats (56, 57). The macropods included in this study demonstrated a larger proportion seropositive to phase I antigen, compared to phase II antigen, with no animals positive for phase II antibody only. The exact mechanism behind this observation is unknown. In contrast, Cooper et al. (11) found an overall higher positivity rate for phase II antibody, compared to phase I antibody, although neither of the two ELISAs used in that study was formally validated and known positive and negative controls were not available. While the pathophysiology and immunophysiology of Q fever and the subsequent immune response, such as the timing of the appearance and persistence of different antibodies, are relatively well studied in humans and domestic animal species (1, 56, 57), these processes are poorly understood in marsupial mammals, complicating the interpretation of test results. Experimental studies and serial sampling following a known infection date or the conduct of a series of cross-sectional studies should be considered to elucidate these pathways in more detail. Based on the findings presented in this study, we recommend that phase I antigen-based tests would be adequate for screening for Q fever for the purpose of serosurveillance in macropods.

Although estimates of the true seroprevalence of C. burnetii seropositivity in the tested macropod populations were not the main aim of this study, the true prevalence estimates generated as part of the latent class analyses provide useful information to support our understanding of the distribution of Q fever in macropods in eastern Australia. The seroprevalence estimates reported here show that the risk of exposure to C. burnetii varied depending on geographical region. This study also highlights some of the highest reported seroprevalence rates in macropod species, with two populations in New South Wales returning estimated true prevalence rates of >80%, adding weight to the existing literature demonstrating that macropods are commonly exposed to C. burnetii and may contribute to the disease ecology of Q fever in both humans and domestic livestock (10, 11, 13). The population with the highest prevalence in this study, at Look At Me Now Headland, is the subject of ongoing health investigations, with nonregenerative anemia and general ill thrift being widespread in the population (36). Whether the high seroprevalence evident in the population is at all related to the ill health of individuals is currently unknown. These points warrant further investigation, including possible public and animal health implications, as well as the role macropods play in the transmission and maintenance cycle of C. burnetii.

Importantly, this study also highlights the necessity of critically evaluating the performance characteristics of diagnostic tests used in a prevalence survey so that estimates of prevalence can be corrected for imperfect diagnostic test performance. There was a significant difference between the true and apparent seroprevalence estimates reported by the ELISA in this study, with the apparent prevalence estimates being markedly lower than the true prevalence estimates. This demonstrates the value of using an appropriately validated test for surveillance programs and serves as a reminder that caution must be exercised when interpreting raw, unadjusted prevalence estimates obtained with unvalidated diagnostic tests.

### Conclusion.

Antibodies to C. burnetii are prevalent in Australian macropods, yet there is large variation in seroprevalence rates among populations in different geographical areas. In order to accurately understand the distribution of coxiellosis in these populations, it is important to use a diagnostic test that has been validated for the target species and has known high diagnostic sensitivity and specificity values. The results of this study show that the IFA, although somewhat more labor-intensive and dependent on skilled interpretation, offers superior diagnostic sensitivity, compared to the commercially available multispecies ELISA, with comparable diagnostic specificity and the additional benefit of being able to distinguish between phase I and phase II antibodies. The IFA would thus be the serological test of choice for screening for the presence of C. burnetii antibodies in kangaroo samples, for example, as part of disease surveillance programs.
