# Supplementary material for: Validation of an Indirect Immunofluorescence Assay and Commercial Q Fever Enzyme-Linked Immunosorbent Assay for Use in Macropods
Source: J Clin Microbiol. 2022 Jun 2;60(7):e00236-22. doi: 10.1128/jcm.00236-22 (PMC9297833; doi:10.1128/jcm.00236-22)
Supplement: Supplemental file 1 — Fig. S1 and S2 and Tables S1 and S2. Download jcm.00236-22-s0001.pdf, PDF file, 0.5 MB [file jcm.00236-22-s0001.pdf]

Supplementary tables and figures - Validation of an indirect immunofluorescence assay and commercial Q fever ELISA for use in macropods

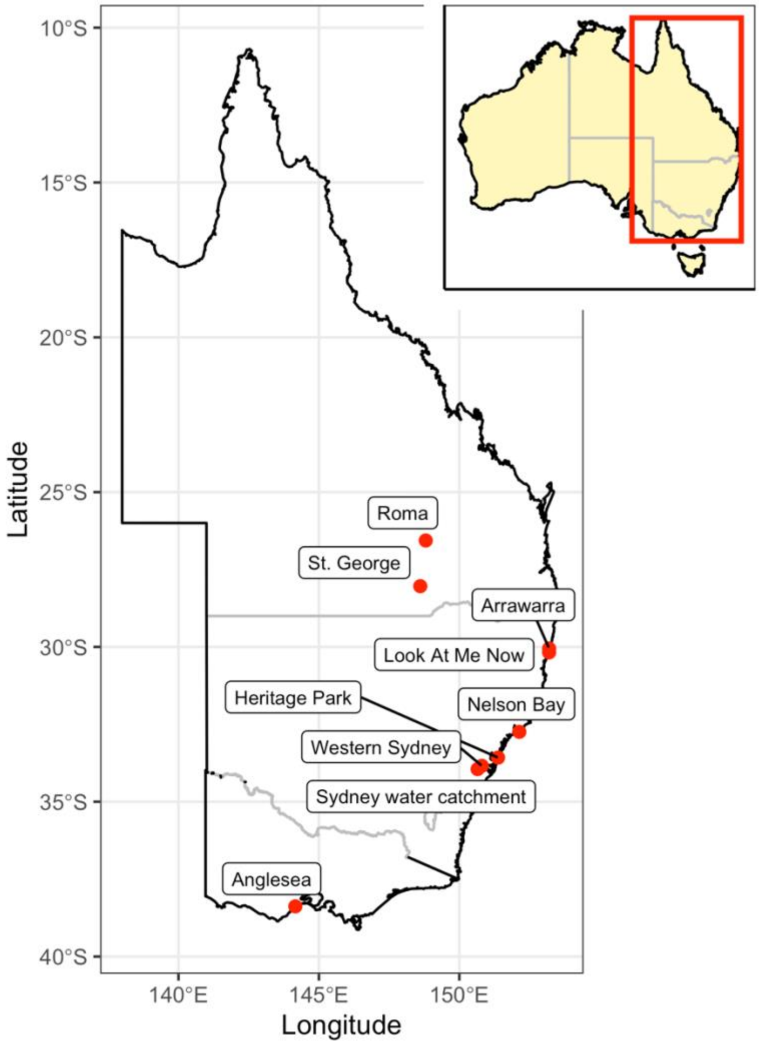

Supplementary Figure 1: Map showing the geographical origins of the Australian macropod samples included in the study.

Supplementary Table 1: Prior distributions used in the Bayesian latent class sensitivity analysis.

Analysis	Parameter	Mode	Lower bound	Beta distribution	
				Alpha	Beta
Baseline:					
IFA	Sensitivity	0.94	0.30	2.608	1.103
IFA	Specificity	0.92	0.35	3.104	1.183
ELISA	Sensitivity	0.90	0.30	2.706	1.190
ELISA	Specificity	0.97	0.35	2.937	1.060
Sydney Basin (NSW)	Prevalence	0.1	0.04	1.114	2.026
Nelson Bay (NSW)	Prevalence	0.2	0.05	1.000	1.000
Coffs Harbour (NSW)	Prevalence	0.2	0.05	1.000	1.000
SW Queensland	Prevalence	0.25	0.05	1.000	1.000
Victoria	Presence	0.05	0.02	2.063	21.197
	Prevalence	0.12	0.04	1.394	3.889
New Zealand	Presence	0.01	0.00	1.335	34.165
	Prevalence	0.01	0.00	1.335	34.165
1. Flat priors for test sensitivity and specificity:					
IFA	Sensitivity	0.5	0.05	1.000	1.000
IFA	Specificity	0.5	0.05	1.000	1.000
ELISA	Sensitivity	0.5	0.05	1.000	1.000
ELISA	Specificity	0.5	0.05	1.000	1.000
Sydney Basin (NSW)	Prevalence	0.1	0.04	1.114	2.026
Nelson Bay (NSW)	Prevalence	0.2	0.05	1.000	1.000
Coffs Harbour (NSW)	Prevalence	0.2	0.05	1.000	1.000
SW Queensland	Prevalence	0.25	0.05	1.000	1.000
Victoria	Presence	0.05	0.02	2.063	21.197
	Prevalence	0.12	0.04	1.394	3.889
New Zealand	Presence	0.01	0.00	1.335	34.165
	Prevalence	0.01	0.00	1.335	34.165
2. Flat priors for the Sydney Basin population:					
IFA	Sensitivity	0.94	0.30	2.608	1.103
IFA	Specificity	0.92	0.35	3.104	1.183
ELISA	Sensitivity	0.90	0.30	2.706	1.190

ELISA	Specificity	0.97	0.35	2.937	1.060
Sydney Basin (NSW)	Prevalence	0.5	0.05	1.000	1.000
Nelson Bay (NSW)	Prevalence	0.2	0.05	1.000	1.000
Coffs Harbour (NSW)	Prevalence	0.2	0.05	1.000	1.000
SW Queensland	Prevalence	0.25	0.05	1.000	1.000
Victoria	Presence	0.05	0.02	2.063	21.197
	Prevalence	0.12	0.04	1.394	3.889
New Zealand	Presence	0.01	0.00	1.335	34.165
	Prevalence	0.01	0.00	1.335	34.165
3. Flat priors for the Victorian population:					
IFA	Sensitivity	0.94	0.30	2.608	1.103
IFA	Specificity	0.92	0.35	3.104	1.183
ELISA	Sensitivity	0.90	0.30	2.706	1.190
ELISA	Specificity	0.97	0.35	2.937	1.060
Sydney Basin (NSW)	Prevalence	0.1	0.04	1.114	2.026
Nelson Bay (NSW)	Prevalence	0.2	0.05	1.000	1.000
Coffs Harbour (NSW)	Prevalence	0.2	0.05	1.000	1.000
SW Queensland	Prevalence	0.25	0.05	1.000	1.000
Victoria	Prevalence	0.5	0.05	1.000	1.000
New Zealand	Presence	0.01	0.00	1.335	34.165
	Prevalence	0.01	0.00	1.335	34.165
4. Flat priors for the New Zealand population:					
IFA	Sensitivity	0.94	0.30	2.608	1.103
IFA	Specificity	0.92	0.35	3.104	1.183
ELISA	Sensitivity	0.90	0.30	2.706	1.190
ELISA	Specificity	0.97	0.35	2.937	1.060
Sydney Basin (NSW)	Prevalence	0.1	0.04	1.114	2.026
Nelson Bay (NSW)	Prevalence	0.2	0.05	1.000	1.000
Coffs Harbour (NSW)	Prevalence	0.2	0.05	1.000	1.000
SW Queensland	Prevalence	0.25	0.05	1.000	1.000
Victoria	Presence	0.05	0.02	2.063	21.197
	Prevalence	0.12	0.04	1.394	3.889
New Zealand	Prevalence	0.5	0.05	1.000	1.000

Supplementary Table 2: Results from the prior sensitivity analysis. Bayesian estimates of the diagnostic sensitivity and specificity for the ELISA and IFA using a range of different priors (1-4) and using the original priors but excluding the red kangaroos and New Zealand (NZ) wallabies (5).

**Analysis**	**ELISA**		**IFA**	
	**DSe (95% CrI)**	**DSp (95% CrI)**	**DSe (95% CrI)**	**DSp (95% CrI)**
1. Flat priors for test parameters	0.415 (0.332 – 0.503)	0.993 (0.964 – 1.000)	0.979 (0.875 – 0.999)	0.988 (0.947 – 1.000)
2. Flat priors for Sydney Basin prevalence	0.421 (0.338 – 0.508)	0.992 (0.966 – 1.000)	0.977 (0.884 – 0.999)	0.985 (0.945 – 0.999)
3. Flat priors for Victorian prevalence	0.422 (0.338 – 0.509)	0.992 (0.962 – 1.000)	0.979 (0.879 – 0.999)	0.985 (0.944 – 0.999)
4. Flat priors for NZ prevalence	0.422 (0.341 – 0.508)	0.992 (0.958 – 1.000)	0.978 (0.887 – 0.999)	0.986 (0.941 – 0.999)
5. Excluding red kangaroos and NZ population	0.386 (0.303 – 0.475)	0.989 (0.946 – 1.000)	0.978 (0.882 – 0.999)	0.976 (0.911 – 0.998)

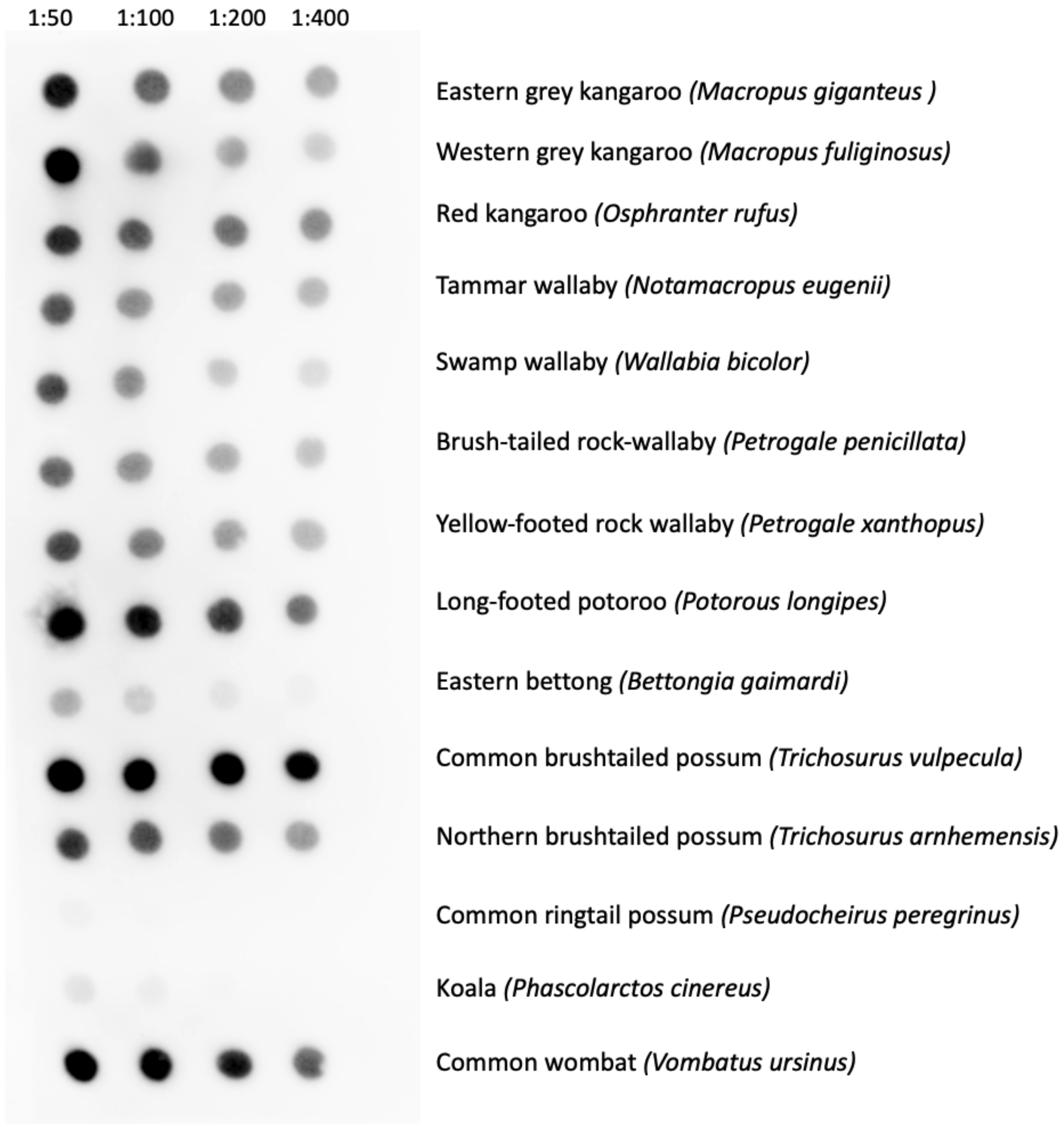

Supplementary Figure 2: Immunoblots of sera from a range of Australian marsupial species against the IDVet ELISA conjugate. Dot blots were performed on serum pools at 1:50, 1:100, 1:200 and 1:400 serum dilutions against a 1:100 conjugate dilution and demonstrates relatively good binding to all macropod species tested.
